# The efficacy of telemedicine interventions on quality of life and depression in individuals with spinal cord injury: a systematic review and meta-analysis

**DOI:** 10.3389/fpsyt.2025.1434376

**Published:** 2025-01-23

**Authors:** YinHu Tan, Xue Liang, Wei Ming, HuiMin Xing, Yan Liang, Yang Wang, Emmanuel Onyebuchi Onodu

**Affiliations:** ^1^ School of Nursing, Changchun University of Chinese Medicine, Changchun, Jilin, China; ^2^ The Affiliated Hospital to Changchun University of Chinese Medicine, Department of Stomatology, Changchun, Jilin, China; ^3^ The Third Affiliated Hospital of Changchun University of Chinese Medicine, Department of Orthopedics, Changchun, Jilin, China

**Keywords:** telemedicine, telerehabilitation, spinal cord injuries, quality of life, depression, meta-analysis

## Abstract

**Objective:**

The purpose of this systematic review and meta-analysis is to examine the impact of telemedicine interventions on the quality of life (QoL) and depression in individuals with spinal cord injury (SCI).

**Methods:**

A literature search was conducted in four electronic databases (PubMed, Web of Science, EMBASE, and the Cochrane Library) from their inception to February 1, 2024. Two authors independently screened the studies and extracted the data. Cochrane’s bias risk tool for randomized controlled trials was used to examine the methodological quality of the included studies. A meta-analysis was conducted using Review Manager (version 5.4) to synthesize the results of the included studies.

**Results:**

A total of 10 trials with 728 participants were included in the review. The results of the meta-analysis showed that telemedicine interventions significantly improved QoL [Standardized Mean Difference (SMD)=0.72, 95% Confidence Interval (CI) (0.11, 1.32), P=0.02] and reduced depression scores [SMD=-0.18, 95% CI (-0.31, -0.05), P=0.006].

**Conclusions:**

Telemedicine interventions are a feasible method to support people with SCI, and can mitigate depression and enhance quality of life. In the future, studies should involve larger sample sizes and extended follow-up periods to validate these findings and to identify the most effective telemedicine interventions for improving the overall health outcomes of individuals with SCI.

**Systematic Review Registration:**

https://www.crd.york.ac.uk/prospero/, identifier CRD42024508702.

## 1 Introduction

SCI is recognized as one of the major clinical challenges of the 21st century, with approximately 500,000 new cases reported worldwide each year (1–3) and a significant increase in incidence among the elderly population (4). Specifically, SCI refers to the structural and functional damage of the spinal cord caused by trauma, disease, or other factors, leading to impairments in motor, sensory, urinary, bowel, and autonomic functions below the level of injury (5), often resulting in permanent paralysis to varying degrees (6). Consequently, post-surgery, people with SCI frequently experience increased pessimism and depression, which diminishes their QoL (7, 8) and imposes a significant economic burden on families and society (9, 10). A study by Van Loo et al. found that 72% of individuals with SCI reported a need for additional care in general, indicating a broad demand for extended support services beyond standard care, which may include but is not limited to telemedicine interventions (11). Telemedicine presents several advantages, including cost savings, enhanced rehabilitation efficiency, and reduced disparities in resource allocation (12). These benefits have led to its widespread adoption for home rehabilitation in people with SCI, particularly following the COVID-19 pandemic (13).

Telemedicine refers to the provision of healthcare services through information and communication technologies, including telephone consultations, video conferencing, web-based platforms, and mobile applications (14). These modalities enable remote monitoring of individuals, consultations, and follow-up care, offering a flexible and accessible alternative to traditional in-person healthcare services. While the effectiveness of telemedicine applications has shown varied results, multiple studies have reported significant positive outcomes for individuals with SCI, particularly in areas such as QoL, functional independence, and care satisfaction. For example, Dallolio et al. (12) found that telemedicine support significantly improved QoL and functional independence for individuals with SCI. In the realm of postoperative pain management, Kolcun et al. (15) observed positive effects from telemedicine, though results for psychological support were mixed. Additionally, Niknamian’s (16) systematic review indicated that telemedicine effectively reduced care costs and enhanced care quality, though results for individuals self-management were variable. Sechrist et al. (17) showed that individuals with SCI experienced significant improvements in satisfaction and rehabilitation outcomes with iPad-based telemedicine, though the effects on long-term psychological support were limited. Similarly, Yuen et al. (18) reported that telemedicine significantly reduced follow-up needs, though psychological support outcomes varied based on individual differences. Additionally, while systematic reviews specifically addressing telemedicine for individuals with SCI are relatively limited compared to other chronic conditions, recent literature has progressively examined its efficacy across diverse health-related concerns within this population. For instance, a systematic review and meta-analysis conducted by Chen et al. (19) demonstrated that telemedicine interventions significantly contribute to the prevention of pressure injuries, which indirectly enhances QoL in individuals with SCI. Rekand et al. (20) investigated the application of remote psychological support for individuals with individuals with SCI, finding that it positively influences the mitigation of depressive symptoms and promotes mental well-being, particularly benefiting those with limited access to in-person psychological services. Furthermore, Mirbaha et al. (21) reviewed various telemedicine service models, underscoring that personalized telemedicine in home care settings markedly improves both QoL and depression management outcomes for individuals with SCI. While these studies elucidate the potential advantages of telemedicine, there remains an absence of systematic reviews and meta-analyses that directly and comprehensively evaluate the effects of telemedicine on overall QoL and depressive symptoms in SCI populations.

Therefore, the objective of this study was to conduct a systematic review and meta-analysis to specifically assess the efficacy of telemedicine interventions in improving quality of life and reducing depression among individuals with SCI, thereby providing further supplementation and evidence to the existing literature.

## 2 Methods

### 2.1 Literature Search

This systematic review and meta-analysis are reported in accordance with the Preferred Reporting Items for Systematic Reviews and Meta-Analyses (PRISMA) 2020 statement (22). A protocol for this review was registered on the PROSPERO International Prospective Register of Systematic Reviews (Registration No. PROSPERO CRD42024508702). We searched the following databases for randomized controlled trials from their inception to February 1, 2024: Web of Science, PubMed, Embase, and the Cochrane Library. Different databases were searched using a combination of MeSH terms, subject terms, and free terms. The following terms were used: telemedicine, telerehabilitation, spinal cord injury, and randomized controlled trial. The specific search strategy is detailed in the **appendix**.

### 2.2 Inclusion Criteria

The inclusion criteria for the study were as follows: (1) Study Design: Randomized controlled trials. (2) Participants: Individuals with spinal cord injury. (3) Interventions: The intervention group received telemedicine interventions, including telephone, application-based, web-based, or other remote technologies, while the control group received routine care for SCI. (4) Outcomes: Reported outcomes including at least one of the following: QoL, depression, anxiety, stress.

### 2.3 Exclusion Criteria

The exclusion criteria for the study were as follows: (1) Individuals with psychiatric disorders and children with SCI. (2) Secondary studies, such as reviews and systematic evaluations. (3) Studies where the full text was not available or original data was missing. (4) Duplicate publications. (5) Conference papers, dissertations, and theses. (6) Non-English papers.

### 2.4 Data Extraction

Endnote X9 software was used to import all references and remove duplicates. The remaining references were independently screened by two researchers according to pre-established inclusion and exclusion criteria. Key information was extracted and cross-checked by both researchers, with any disagreements resolved through discussion or by involving a third researcher. The extracted data included author’s name, year of publication, country, sample size, mean age, research focus, injury duration, injury level, injury etiology, interventions in the experimental and control groups, follow-up duration, and outcome measures.

### 2.5 Quality Assessment

The risk of bias in the included studies was assessed by two researchers independently using the Cochrane Handbook for Systematic Reviews of Interventions (23). Studies with evident design biases were excluded. The assessment tool covered the following seven domains: random sequence generation, allocation concealment, blinding of participants and personnel, blinding of outcome assessors, incomplete outcome data, selective reporting, and other sources of bias. Each domain was rated as “low risk of bias,” “high risk of bias,” or “unclear.” Any disagreements in the assessment were resolved through discussion or by involving a third researcher, resulting in a consensus.

### 2.6 Statistical Analysis

This study employed Review Manager 5.4 software for conducting meta-analyses. When outcome measures were consistent across studies, the effect size was calculated as the mean difference (MD) with corresponding 95% confidence intervals (CIs). For outcome measures with variability, such as differences in assessment tools or measurement scales, standardized mean differences (SMD) were used to facilitate comparability. Heterogeneity among the included studies was assessed using the I² statistic, which measures the proportion of variability across studies attributable to heterogeneity rather than chance. An I² value exceeding 50% indicated substantial heterogeneity, warranting the use of a random-effects model with the DerSimonian and Laird method as implemented in RevMan. For I² values below 50%, a fixed-effects model with the Mantel-Haenszel method was used. Subgroup analyses were performed to investigate sources of heterogeneity based on key study characteristics, including intervention type and duration. A funnel plot was created to visually assess publication bias. Symmetry in the funnel plot indicated a low risk of publication bias, whereas asymmetry suggested potential publication bias or small-study effects. An asymmetrical funnel plot, with missing studies on one side, could suggest selective reporting of significant results. Given the small number of included studies (fewer than 10), the statistical power to detect asymmetry was low, necessitating cautious interpretation of the results.

## 3 Results

### 3.1 Literature Search

**Figure 1:**
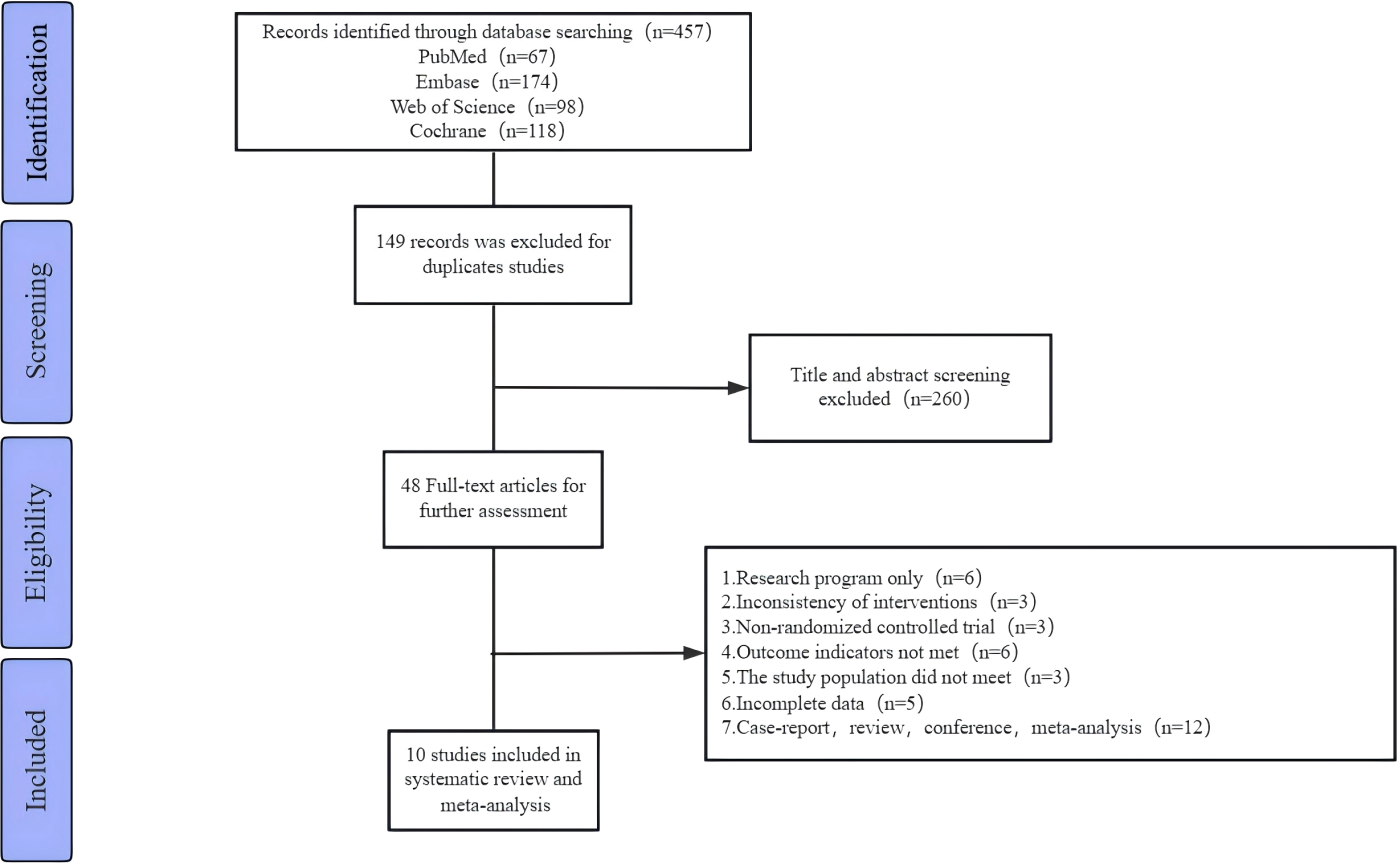
Flowchart of study selection.

The PRISMA (Preferred Reporting Items for Systematic Reviews and Meta-Analyses) flow diagram for this study is presented in **Figure 1**. The search strategy yielded 457 articles from four databases. After removing duplicates and screening titles and abstracts, 48 articles were retained for full-text evaluation. Based on the inclusion and exclusion criteria, only 10 studies (6, 24–32) were included in this meta-analysis. All of these studies were randomized controlled trials published between 2012 and 2023.

### 3.2 Risks of Bias and Quality Assessment

**Figure 2:**
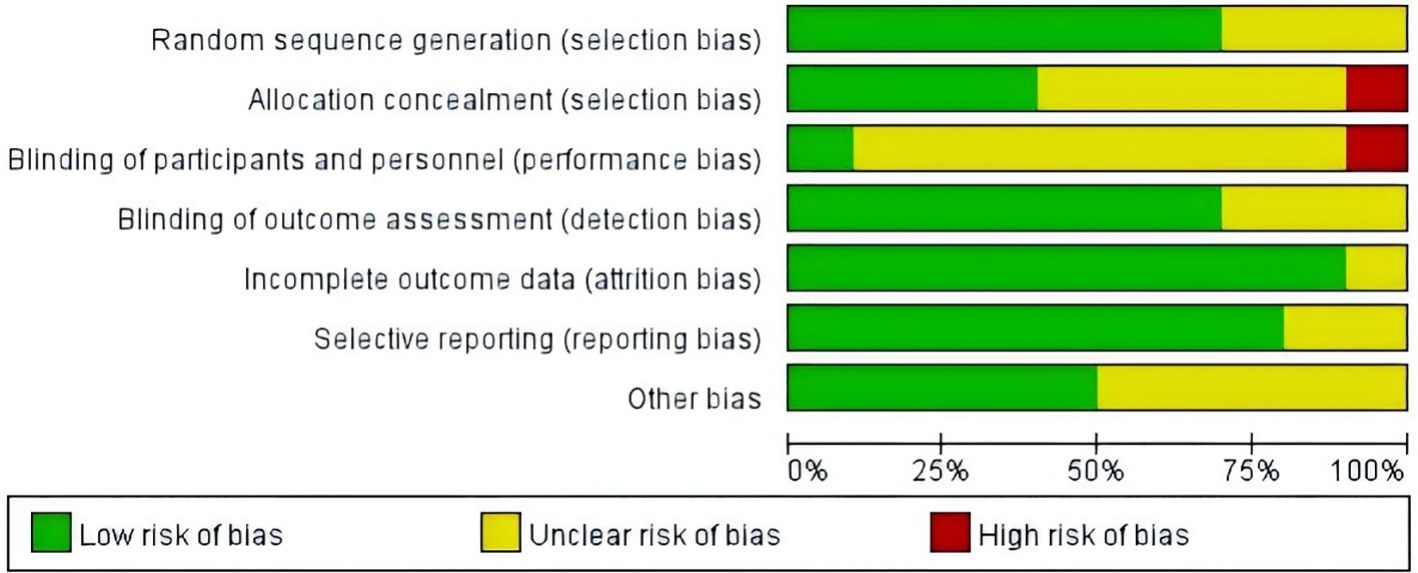
Risk of bias graph.

**Figure 3:**
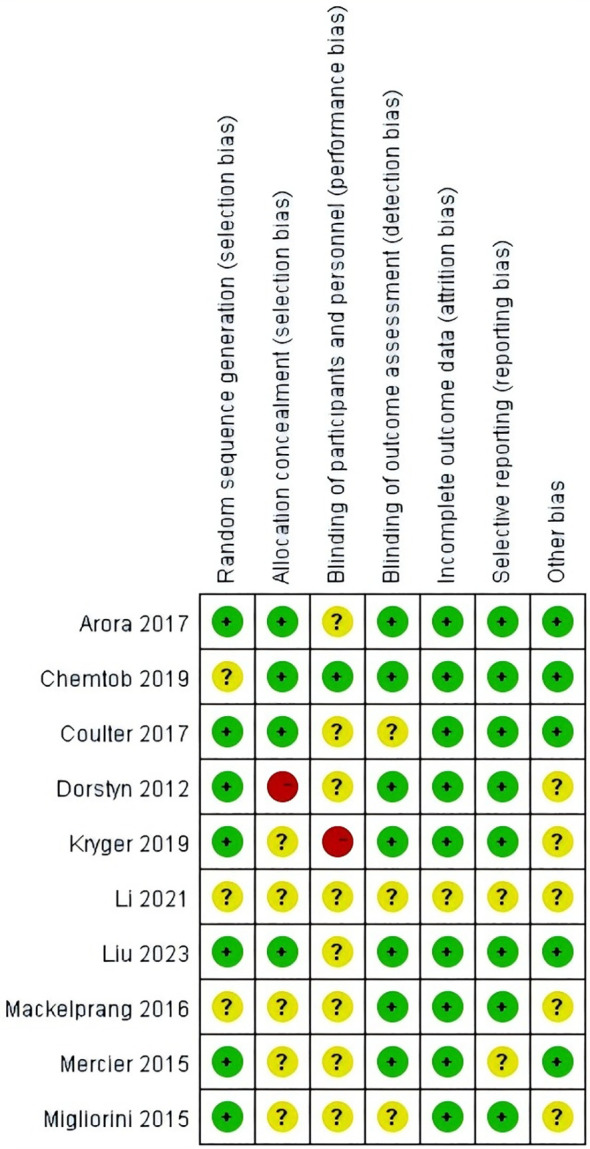
Risk of bias summary.

According to the Cochrane Handbook’s assessment of bias risk in the included studies, the results indicate that a high risk of bias was present in only a few areas, while most criteria were met satisfactorily, suggesting that the overall quality of the included studies was moderate (33). Seven studies (24, 26–29, 31, 32) described the methods of random sequence generation, while three studies (6, 25, 30) mentioned randomization without providing specific details. Additionally, four studies (24–26, 29) implemented allocation concealment, indicating a lower risk of bias. Regarding detection bias, one study (25) employed double-blinding for both participants and assessors. In eight studies (6, 24, 26, 27, 29–32), it was unclear whether blinding was applied to participants, and in three studies (6, 26, 32), it was unclear whether assessors were blinded. One study (28) exhibited a high risk of bias concerning the blinding of participants or assessors. The risk of bias for incomplete outcome data, selective reporting, or other biases was generally low across the 10 included studies (6, 24–32).Detailed risk of bias assessments and summaries are presented in **Figures 2** and **3**.

### 3.3 Study and Participant Characteristics

Among the 10 included studies (6, 24–32), there were a total of 728 participants, with an average age ranging from 40 to 60 years. These studies were published between 2012 and 2023 and were conducted in seven countries, including the United States (n=3) (28, 30, 31), the United Kingdom (n=1) (26), Australia (n=2) (27, 32), Canada (n=1) (25), China (n=2) (6, 29), India and Bangladesh (n=1) (24). Five studies (24, 27, 28, 30, 31) utilized telephone-based remote interventions, Five studies (25, 26, 29, 32) employed web-based or app-based online learning. In the study by Hearn et al., the remote intervention was delivered via a webpage, enabling participants to engage in mindfulness training through audio sessions provided on the site. However, the article did not specify the website address or the name of the webpage. Chemtob et al. (25) utilized the REACTS^®^ video software for interventions, which provided counseling sessions aimed at enhancing motivation for recovery and participation in physical exercise through behavior change techniques and self-regulation strategies. Coulter et al. (26) employed a self-developed website (www.webbasedphysio.com) for remote personalized physical therapy, enabling individuals to complete rehabilitation exercises tailored to their specific exercise plans, thereby better meeting each individual’s needs. One study utilized SurveyMonkey^©^ software, which provided online learning packages for remote education, helping individuals with SCI find employment, facilitate reintegration into society, and reduce anxiety. Liu et al. (29) utilized an app called ‘Together,’ which included post-discharge health education and follow-up services. Individuals could use the app to interact with researchers via video, phone calls, and text messages to address any issues. The team led by Migliorini et al. (32) developed the Electronic Personal Administration of Cognitive Therapy (ePACT) program, which included a 10-module skills and psychological education plan aimed at improving negative emotions through structured learning. One study (6) utilized an online home care intervention that included video education and an internet platform built using WeChat, a mobile app, telephone, Microblog, and QQ groups. This platform provided multi-channel access to consultation services for care, medical treatment, prevention, and health maintenance. Additionally, researchers could publish disease-related information on the platform for individuals to access and learn from, and they could also engage in one-on-one conversations with the researchers. The follow-up duration in the included studies ranged from 4 weeks to 12 months.

The degree of depression was assessed using various scales, including the Depression Anxiety Stress Scale-21 (DASS-21), Patient Health Questionnaire-9 (PHQ-9), the Hospital Anxiety and Depression Scale (HADS), and Beck Depression Inventory-II (BDI-II), with lower scores indicating less severe depression. The QoL was evaluated using the Delighted-Terrible Scale (DTS), The World Health Organization Quality of Life Brief Scale (WHO-QOL BREF), Euro Quality of Life 5-Dimensional 5-Level (EQ-5D-5L), The Life Satisfaction Questionnaire-11 (LSQ-11), and The MOS 36-Item Short Form Health Survey (SF-36), with higher scores indicating better QoL. Additionally, anxiety was assessed using the HADS and DASS-21 subscales, while stress was evaluated using the DASS-21 subscale. Detailed characteristics of the included studies are presented in **Tables 1**–**3**.

**Table 1:** Basic characteristics of the study.

Author/year	Country	Participants		Age	Intervention group		Control Group		Outcomes assessment time	Outcomes (outcome measures)
		T	C		Intervention methods	Frequency & duration	Intervention methods	Frequency & duration		
Dorstyn2012 (27)	Australia	20	19	I: 53.8 ± 16.3 C: 53.1 ± 20.0	Telephone counseling: the introduction of interventions, education on the psychosocial impact of SCI, education, reinforcement, and monitoring of positive coping strategies, and referral to community psychological services when needed.	Once every two weeks, each time less than 20 minutes	Standard care: routine individual medical follow-up and physical therapies (eg, physiotherapy, occupational therapy), in addition to a face-to-face consultation with a psychologist (D.D.) at 3 months post discharge.	Not mention	Baseline, 3m, 6m	Depression, Anxiety, Stress (DASS-21)
Mercier2015 (31)	USA	53	53	I: 45.8 ± 12.1 C: 45.0 ± 14.0	Telephone counseling: Regular automated calls provide educational content related to depression, skincare, and the perspectives of peers and clinical experts + CareCall Resource Book	Once a week for 3 months and once every two weeks after 3 months for 3 months	Standard care + CareCall Resource Book	Not mention	Baseline, 6m	Depression (PHQ-9)
Migliorini 2016 (32)	Australia	23	25	I: 47.5 ± 12.2 C: 52.8 ± 12.9	Online learning: A 10-module skills and psycho-educational program based primarily on cognitive behavioral therapy principles, but with added aspects of positive psychology and mindfulness meditation	one module per week	Wait-list control	Not mention	Baseline, 3m, 6m	Depression, Anxiety, Stress (DASS-21)
Mackelprang 2016 (30)	USA	85	83	I: 40.4 ± 15.7 C: 42.0 ± 16.0	Telephone counseling: (Education, problem-solving, referral resources and support) +usual care	At 1, 2, 3 and 6 weeks and 2, 3, 4, 6, 8 and 10 months after discharge, 30-45 minutes each time	Usual care (standard referrals and follow-up)	Not mention	Baseline, 12m	QoL (DTS) Depression (PHQ-9)
Coulter 2017 (26)	Britain	15	6	I: 51.5 ± 13.0 C: 48.1 ± 10.6	Online learning: The website consists of exercise, exercise diary, advice and education sections, the physical therapist can create a personalized exercise plan, remotely view the exercise diary, and change the exercise plan	30 minutes at least twice a week for 8 weeks	Usual care: consisting of self-management of their condition. If participants were currently exercising (for example, home-based exercise, gym or exercise class), they were asked to continue and to keep an exercise diary noting any exercise or activities in which they participated.	Not mention	Baseline, 8w	QoL (WHO-QOL BREF) Depression, Anxiety (HADS)
Arora 2017 (24)	India, Bangladesh	57	58	I: 35.0 ± 11.0 C: 36.0 ± 12.0	Telephone counseling: Get a pamphlet containing information on pressure ulcer management, call a trained healthcare professional, reinforce self-help strategies important for managing pressure ulcers, minimizing psychological stress, and increasing life engagement	1 time per week	Release of information kit (pressure ulcer management at the time of recruitment, and were free to seek any help or medical assistance that they deemed appropriate or could access.)	Not mention	Baseline, 12w	QoL (EQ-5D-5L) Depression (HADS)
Kryger 2019 (28)	USA	16	17	I: 37.9 ± 13.4 C: 44.1 ± 15.3	Online learning: The app includes medication management, urination, and defecation schedule reminders, photo-enabled skincare tracking, mood tracking, and messaging	Not mention	Standard care (Intermittently attended by a physician in an outpatient setting and followed up as needed. Nurses talk to individuals on the phone offer advice and relay concerns to physicians)	Not mention	Baseline, 3m, 6m, 9m	Depression (BDI-II)
Chemtob 2019 (25)	Canada	10	12	A: 51.6 ± 12.1	Online learning: An online video chat platform conducts counseling sessions designed to foster recreational physical activity (LTPA) motivation and participation through the use of behavior change techniques and self-regulatory strategies	once a week for 1 hour each time for 8 weeks	Regular daily routine (Continue their daily activities and are not encouraged to increase or decrease their current level of physical activity)	Not mention	Baseline, 4w, 8w	QoL (TLSQ-11) Depression (PHQ-9)
Li 2021 (6)	China	40	40	I: 57.8 C: 59.1	Online learning: 1. Establish the individuals with TSCI individuals database; 2. Conduct video health education; 3. Internet platforms push knowledge	Not mention	Routine discharge guidance	Not mention	Baseline, 12m	QoL (SF-36)
Liu 2023 (29)	China	49	49	I:40.37 ± 12.18C:43.06 ± 12.06	Online learning: The APP includes discharge health education (information sheets of individuals and a list for discharge education) and follow-up visits (online assessment, health education, interdis-ciplinary referral, and interaction)	5 one-on-one online sessions were scheduled at the 2nd, 4th, 6th, 8th, and 12th weeks after discharge.	Routine counselling (Daily care included health education and telephone follow-up by the visiting nurse at 12 weeks after discharge) +Health education CD	Not mention	Baseline, 12w,24w	Depression (BDI-II)

I, Intervention group; C, Control group; A, All groups; DASS-21, Depression Anxiety Stress Scale-21; PHQ-9, Patient Health Questionnaire-9; EPACT, Electronic Personal Administration of Cognitive Therapy; DTS, Delighted-Terrible Scale; WHO-QOL BREF, The World Health Organisation Quality of Life Bref Scale; HADS, The Hospital Anxiety and Depression; EQ-5D-5L, Euro Quality of Life 5-dimensional 5-level; BDI-II, Beck Depression Inventory-II; TLSQ-11, The Life Satisfaction Questionnaire-11; SF-36, The MOS 36-Item Short Form Health Survey.

**Table 2:** Participant Characteristics and Study Focus in Telemedicine Interventions for SCI.

Author/year		Injury Duration	Injury Level	Injury Etiology
Dorstyn2012 (27)	Evaluates the impact of telephone counseling on mental health and life satisfaction in recently injured SCI individuals	Recently injured, less than 1 year	Paraplegia: 62%; Tetraplegia: 38%	Traumatic: 56%; Non-traumatic: 44%
Mercier2015 (31)	Explores the impact of the CareCall system on health management among individuals with SCI	Average 11.8 years	Cervical (C1-C8): 48.1%; Thoracic (T1-T12): 43.4%; Lumbar (L1-L2): 5.7%	Not specified
Migliorini 2016 (32)	Assesses the effects of ePACT electronic cognitive behavioral therapy on depressive symptoms and life satisfaction in individuals with SCI	Intervention group: 11.4 years, Control group: 19.8 years	Complete tetraplegia: 3% (intervention), 8% (control); Incomplete tetraplegia: 53% (intervention), 8% (control); Complete paraplegia: 15% (intervention), 28% (control); Incomplete paraplegia: 24% (intervention), 52% (control)	Includes both traumatic and non-traumatic injuries
Mackelprang 2016 (30)	Studies the impact of telephone consultations on health service use and mental health in individuals with SCI	Recently injured (within the first year post-rehabilitation)	Cervical (C1-C4): 20%; Cervical (C5-C8): 13%; Paraplegia: 28%; AIS D (incomplete): 38%	Motor vehicle accident: 35%; Violence: 8%; Sports/recreation: 14%; Falls/struck by object: 37%; Other: 5%
Coulter 2017 (26)	Assesses online physical therapy’s impact on health and satisfaction in individuals with SCI	Intervention group: 13 years (SD 11.6 years), Control group: 15.7 years (SD 9.7 years)	Cervical (C1-C4): 6% (intervention), 13% (control); Cervical (C5-C8): 25% (intervention), 63% (control); Thoracic-sacral (T1-S5): 69% (intervention), 25% (control)	Complete injury: 44% (intervention), 63% (control); Incomplete injury: 56% (intervention), 38% (control)
Arora 2017 (24)	Examines the effectiveness of telephone-based intervention for pressure injury management in individuals with SCI in low- and middle-income countries	Average 7 years (SD 13 years)	Upper cervical (C1-C4): 2% (intervention), 7% (control); Lower cervical (C5-C8): 19% (intervention), 22% (control); Upper thoracic (T1-T9): 28% (intervention), 23% (control); Lower thoracic (T10-L1): 35% (intervention), 38% (control); Lumbar-sacral (L2-S5): 3% (intervention), not reported in control group	Motor vehicle accidents: 53% (intervention), 38% (control); High falls: 30% (intervention), 32% (control); Other: 17% (intervention), 25% (control)
Kryger 2019 (28)	Evaluates the iMHere mHealth system’s impact on health and psychosocial outcomes in individuals with SCI	Intervention group: 9.9 years (SD 8 years), Control group: 13.5 years (SD 11 years)	Paraplegia: 58% (intervention), 53% (control); Tetraplegia: 42% (intervention), 47% (control)	Complete injury: 47% (intervention), 63% (control); Incomplete injury: 53% (intervention), 37% (control)
Chemtob 2019 (25)	Assesses the impact of self-determination theory-based telehealth intervention on leisure physical activity and quality of life in individuals with SCI	Minimum injury duration of 1 year	Limited to paraplegia, below cervical level	Etiology not reported
Li 2021 (6)	Explores the effect of an online family care model on functional impairment, complications, and quality of life in traumatic individuals with SCI	Includes 1-year follow-up post-discharge	Cervical, thoracic, and lumbar injuries	Traumatic, including motor vehicle accidents and falls
Liu 2023 (29)	Evaluates the effects of an APP-based self-management intervention on depression in community-dwelling individuals with SCI	Injury duration of 2 years or less	Cervical: 29.6%, Thoracic: 46.9%, Lumbar: 23.5%; Includes both complete and incomplete injuries	Primarily traumatic (90.8%)

**Table 3:** Summary of Included Studies: Primary and Secondary Outcome Measures.

First Author/Year	Sample Size and Population	Primary Purpose	Primary Outcome	Secondary Outcome
Dorstyn2012 (27)	48, SCI	To evaluate the impact of remote psychological interventions on emotional regulation in individuals with SCI	Depression, anxiety, stress	Emotional health and social support
Mercier2015 (31)	106, SCI 36, MS	To assess the effectiveness of telemedicine in individuals with SCI and MS	Depression, skin integrity, healthcare utilization, participation, satisfaction	Quality of life
Migliorini 2016 (32)	48, SCI	To evaluate the impact of remote cognitive behavioral therapy on emotional health and quality of life in individuals with SCI	Emotional health, life satisfaction	Depression, anxiety, stress
Mackelprang 2016 (30)	100, SCI	To assess the impact of telephone counseling on healthcare utilization and mental health in individuals with SCI	Healthcare utilization	Depression, quality of life, current health status, subjective health
Coulter 2017 (26)	150, SCI	To assess the effectiveness and participant satisfaction with remote rehabilitation training in individuals with SCI	Motor function, quality of life	Depression, anxiety
Arora 2017 (24)	120, SCI	To evaluate the effectiveness of remote telephone intervention on pressure ulcer healing in individuals with SCI	Pressure ulcers	Pain reduction, depression
Kryger 2019 (28)	75, SCI	To assess the effectiveness of video-based cognitive behavioral therapy on mental health in individuals with SCI	Independence, depression, quality of life	N/A
Chemtob 2019 (25)	50, SCI	To assess the satisfaction, physical activity, and quality of life improvements from telemedicine in individuals with SCI	Psychological need satisfaction	Physical activity, quality of life, depression, daily activity participation
Li 2021 (6)	60, SCI	To explore the application of a network-based home care model in individuals with SCI	Complications incidence, functional disability, quality of life	N/A
Liu 2023 (29)	500, SCI	To evaluate the impact of an APP-based self-management intervention on depression and anxiety in individuals with SCI	Depression, anxiety	Life quality change assessment

### 3.4 Primary Outcomes

#### 3.4.1 QoL

**Figure 4:**
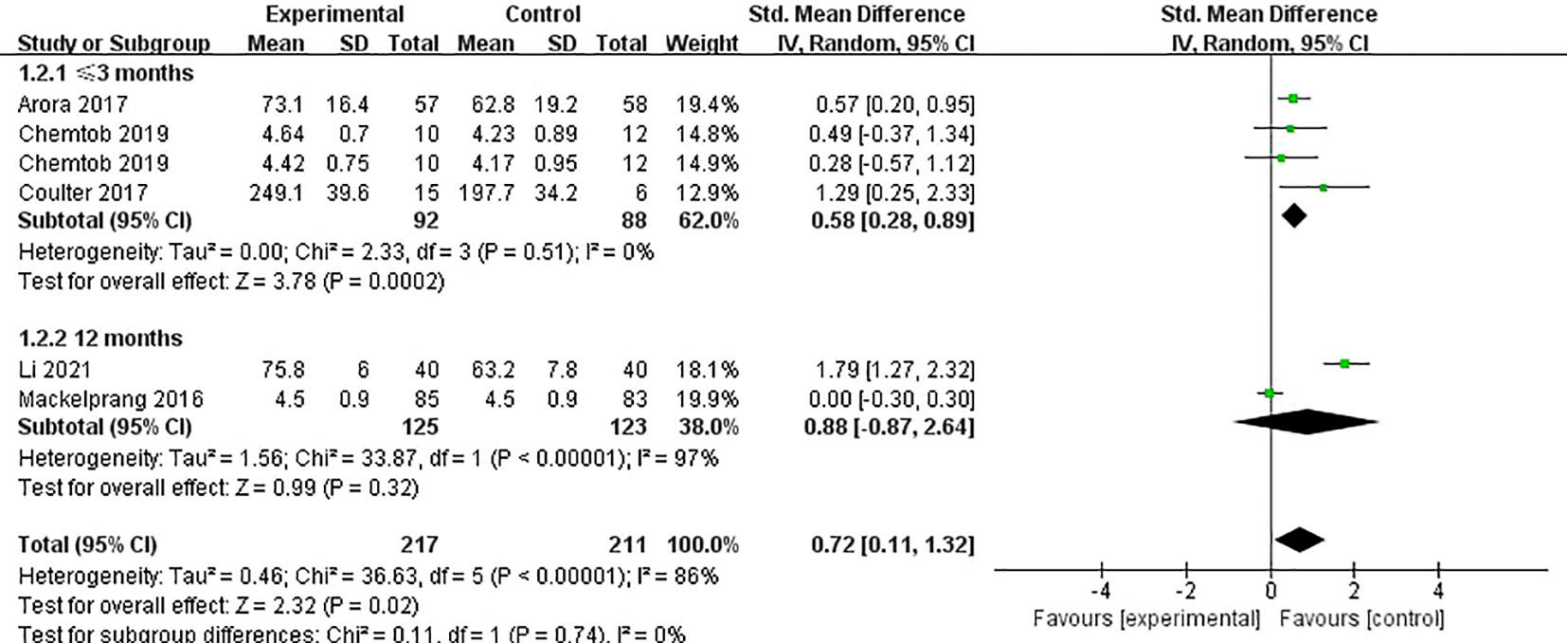
Forest plot: effect of different follow-up times on QoL.

**Figure 5:**
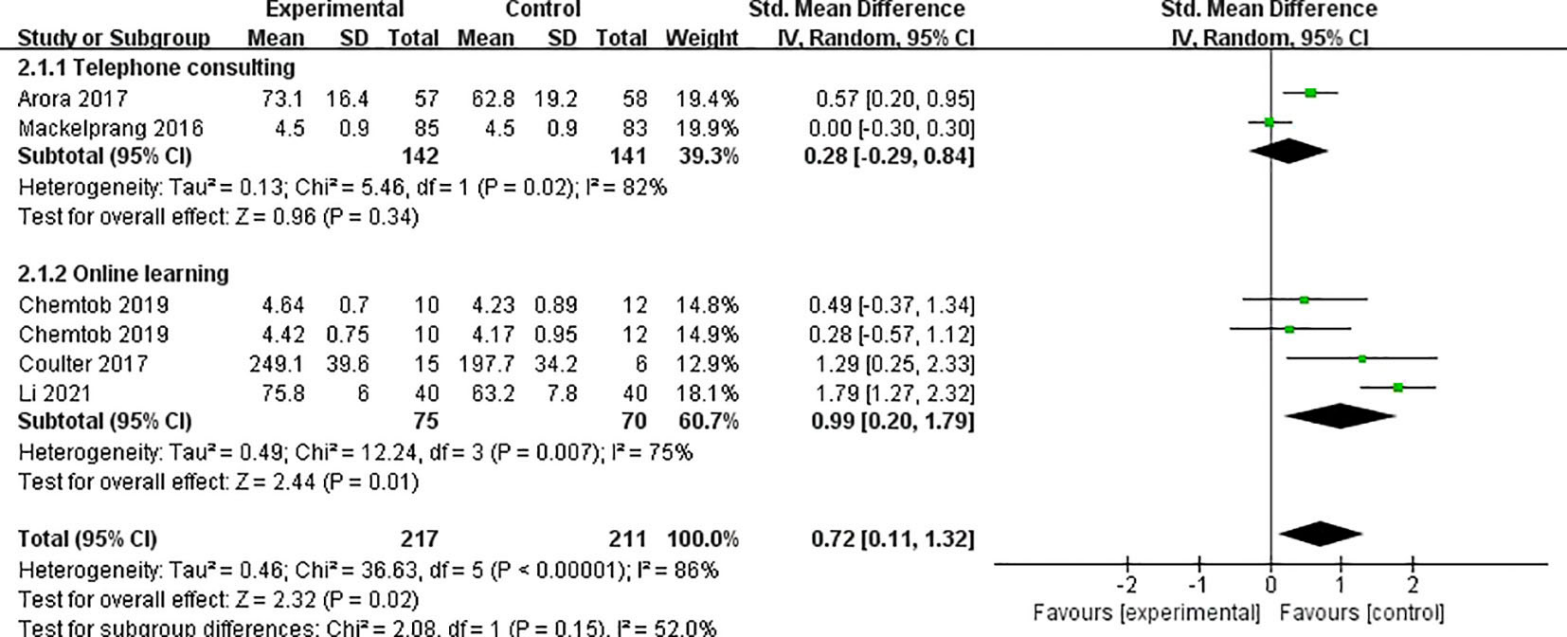
Forest plot: effect of different interventions on QoL.

Five studies (6, 24–26, 30) reported on the impact of telemedicine on the quality of life (QoL) of individuals with SCI, with high heterogeneity observed among the studies (*P*<0.0001, I²=86%), likely due to the use of different scales to assess QoL in each study. Therefore, a random-effects model was applied for the analysis. The results indicated that telemedicine significantly improved the QoL for individuals with SCI compared to the control group [SMD=0.72, 95% CI (0.11, 1.32), *P*=0.02]. Subgroup analyses based on follow-up time and intervention method revealed that within 3 months, telemedicine interventions significantly improved the QoL for individuals with SCI compared to the control group [SMD=0.58, 95% CI (0.28, 0.89), *P*=0.0002], while no significant difference was observed at 12 months [SMD=0.88, 95% CI (-0.87, 2.64), *P*=0.32] (**Figure 4**). Telephone-based telemedicine showed no significant difference compared to the control group [SMD=0.28, 95% CI (-0.29, 0.84), *P*=0.34], whereas online learning-based telemedicine showed a significant improvement in QoL compared to the control group [SMD=0.72, 95% CI (0.11, 1.32), *P*=0.01] (**Figure 5**).

#### 3.4.2 Depression

**Figure 6:**
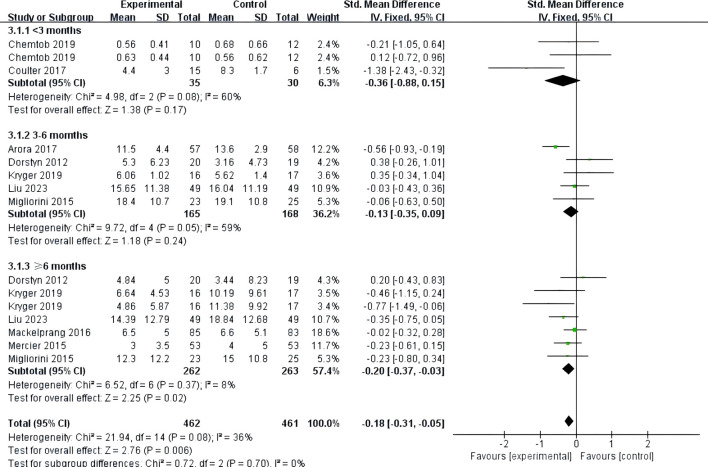
Forest plot: effect of different follow-up times on depression.

**Figure 7:**
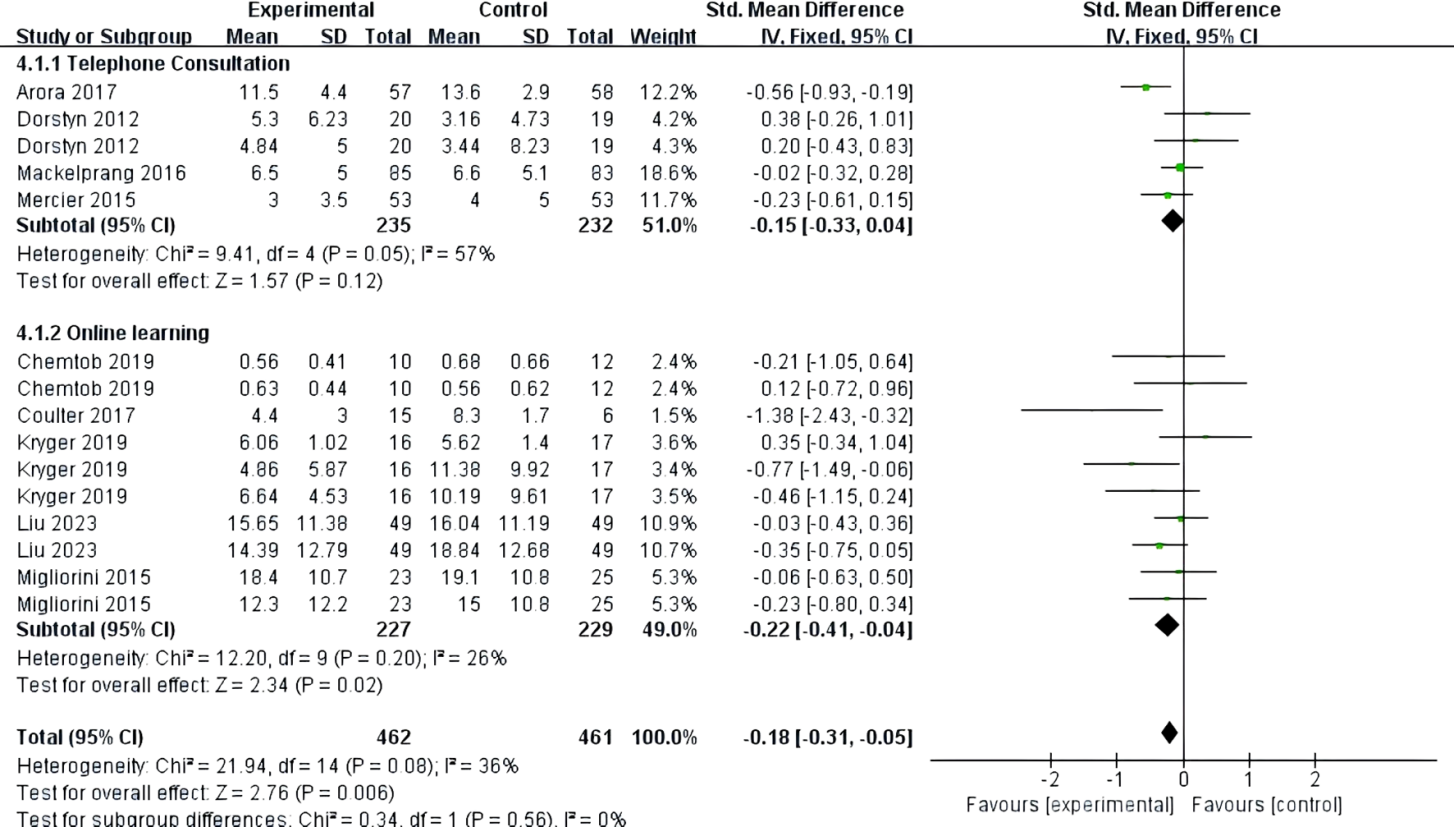
Forest map: effect of different interventions on depression.

Nine studies (24–32) reported on the impact of telemedicine on depression in individuals with SCI. The heterogeneity among these studies was low (*P*=0.08, *I*²=36%), so a fixed-effects model was used for the analysis. The results indicated that telemedicine significantly improved depression in individuals with SCI compared to the control group [SMD=-0.18, 95% CI (-0.31, -0.05), *P*=0.006]. Subgroup analyses based on follow-up time and intervention method showed that within 3 months, there was no significant difference in the improvement of depression between the telemedicine group and the control group [SMD=-0.36, 95% CI (-0.88, 0.15), *P*=0.17], and between 3-6 months, there was also no significant difference [SMD=-0.13, 95% CI (-0.35, 0.09), *P*=0.24]. However, at 6 months and beyond, there was a significant difference in the improvement of depression between the telemedicine group and the control group [SMD=-0.20, 95% CI (-0.37, -0.03), *P*=0.02] (**Figure 6**). For intervention methods, telephone-based telemedicine showed no significant difference compared to the control group [SMD=-0.15, 95% CI (-0.33, 0.04), *P*=0.12], whereas online learning-based telemedicine showed a significant improvement in depression compared to the control group [SMD=-0.22, 95% CI (-0.41, -0.04), *P*=0.02] (**Figure 7**).

### 3.5 Secondary Outcome

#### 3.5.1 Anxiety

**Figure 8:**
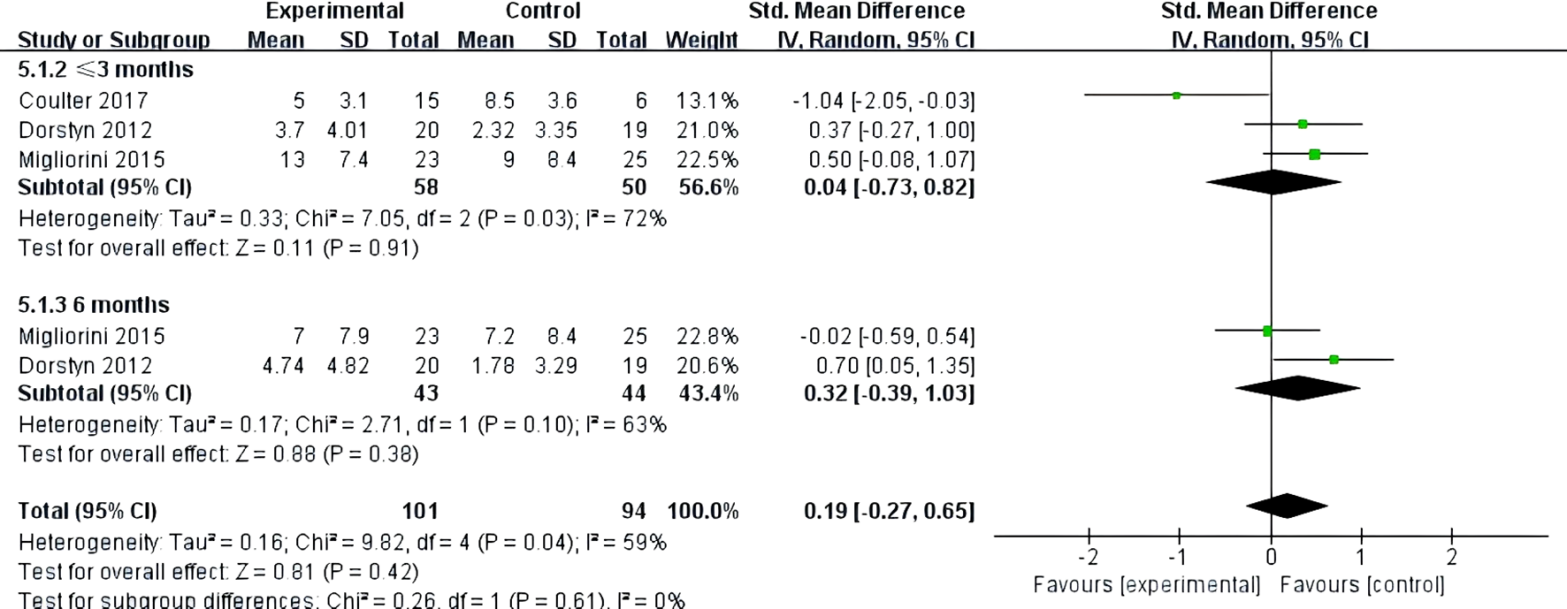
Forest plot: effect of different follow-up times on anxiety.

Three studies (26, 27, 32) reported on the impact of telemedicine on anxiety in individuals with SCI. Among them, one study (26) used the HADS-A subscale of the HADS to assess anxiety, while the other two (27, 32) used the anxiety subscale of the DASS-21. The heterogeneity among these studies was high (*P*=0.04, *I*²=59%), so a random-effects model was used for the analysis. The results indicated that there was no significant difference in the improvement of anxiety between the telemedicine group and the control group [SMD=0.19, 95% CI (-0.27, 0.65), *P*=0.42]. Subgroup analysis based on follow-up time showed that within 3 months [SMD=0.04, 95% CI (-0.73, 0.82), *P*=0.91] and at 6 months [SMD=0.32, 95% CI (-0.39, 1.03), *P*=0.38], there were no significant differences in the improvement of anxiety between the telemedicine group and the control group (**Figure 8**).

#### 3.5.2 Stress

**Figure 9:**
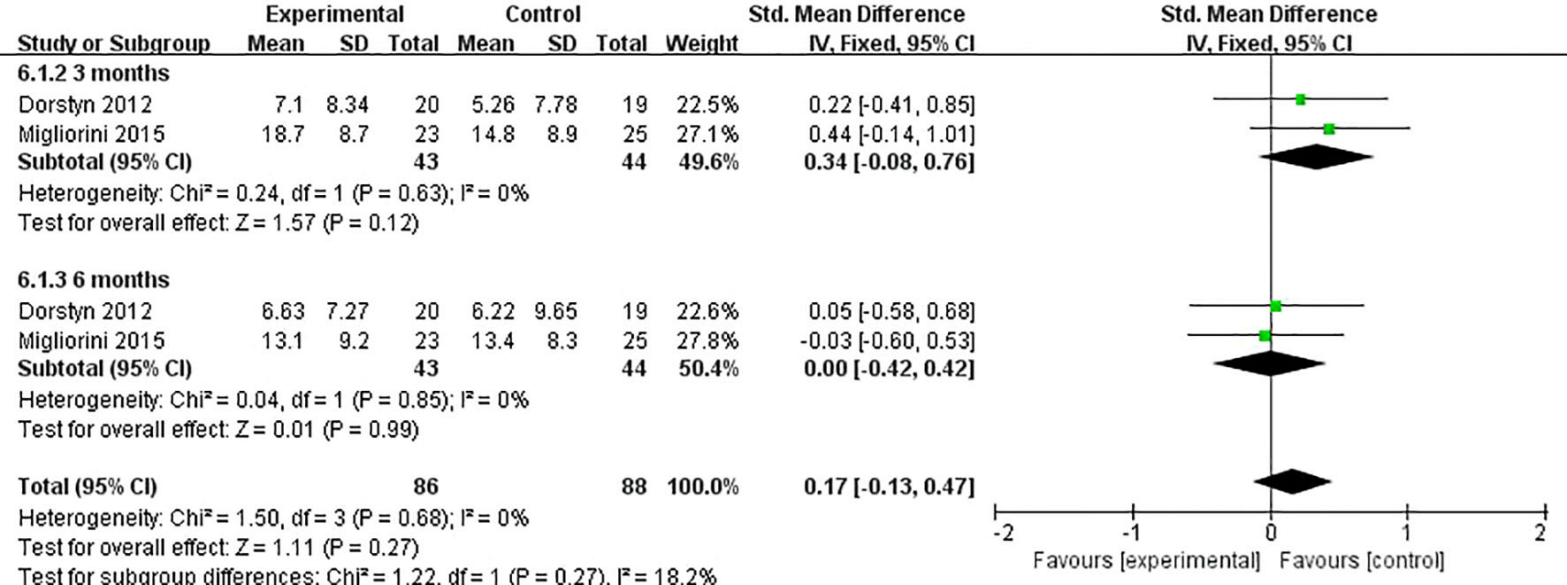
Forest plot: effect of different follow-up times on stress.

Two studies (27, 32) reported on the impact of telemedicine on stress in individuals with SCI. Both studies used the stress subscale of the DASS-21 for assessment. The heterogeneity among these studies was low (*P*=0.68, *I*²=0%), so a fixed-effects model was used for the analysis. The results indicated that there was no significant difference in the improvement of stress between the telemedicine group and the control group [SMD=0.17, 95% CI (-0.13, 0.47), *P*=0.27]. Subgroup analysis based on follow-up time showed that at 3 months [SMD=0.34, 95% CI (-0.08, 0.76), *P*=0.12] and at 6 months [SMD=0.00, 95% CI (-0.42, 0.42), *P*=0.99], there were no significant differences in the improvement of stress between the telemedicine group and the control group (**Figure 9**).

### 3.6 Sensitivity Analysis and Publication Bias

**Figure 10:**
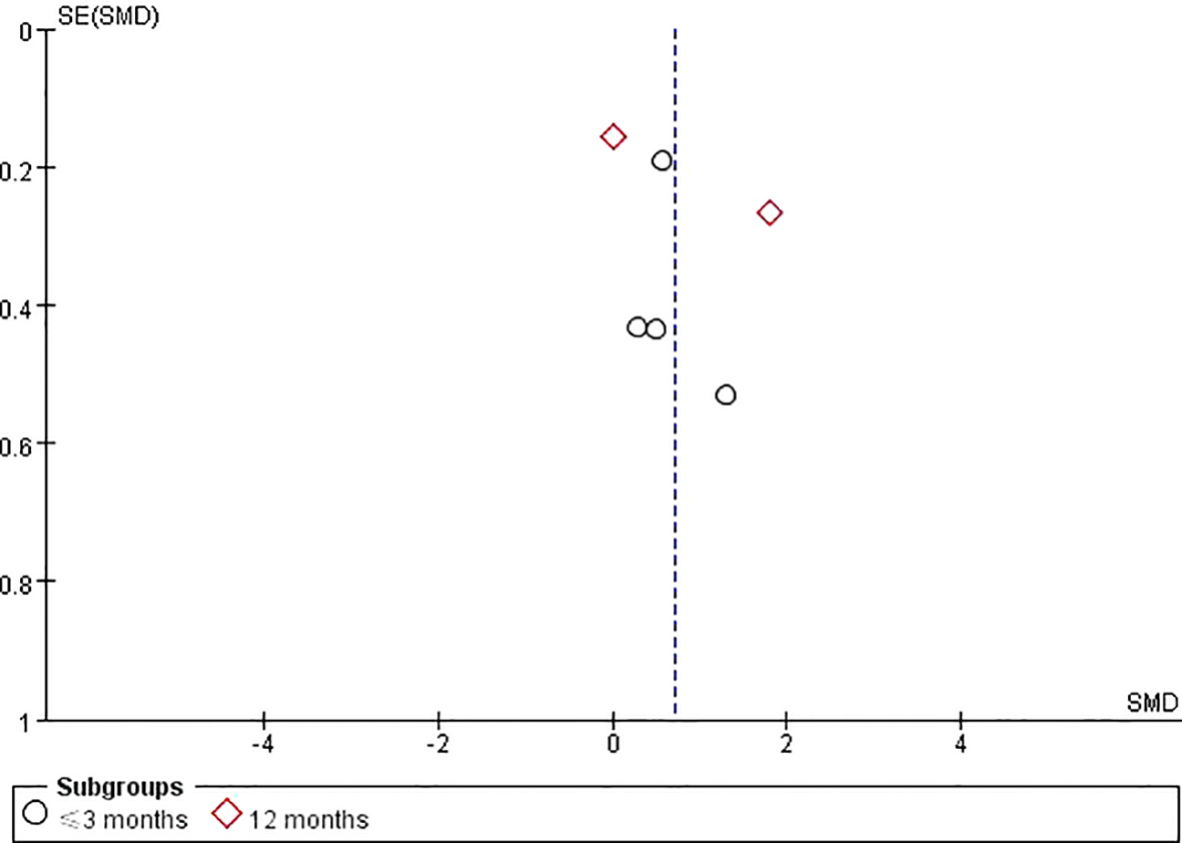
QoL funnel plot.

**Figure 11:**
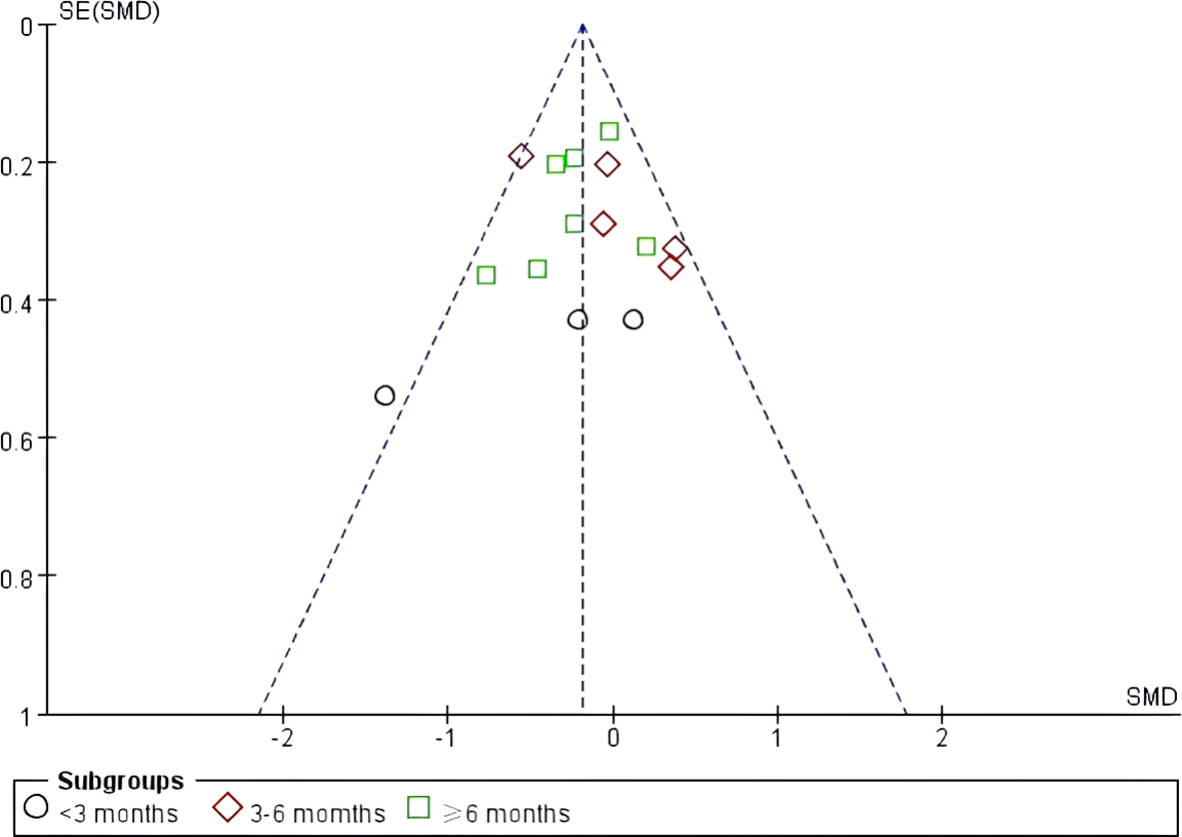
Depression funnel plot.

A sensitivity analysis was conducted by sequentially removing each of the 10 included studies (6, 24–32), and the results showed no significant changes, indicating that the findings of this study are relatively stable. Funnel plots were generated for the primary outcomes, QoL and depression in individuals with SCI. The results showed that the funnel plots were approximately symmetrically distributed, suggesting a low likelihood of publication bias among the included studies (**Figures 10**, **11**).

## 4 Discussion

### 4.1 Main Outcome Indicators

#### 4.1.1 Effectiveness of Telemedicine on QoL in Individuals with SCI

The results of this study indicate that telemedicine can significantly improve the QoL of individuals with SCI, with a statistically significant difference (*P*<0.05). Although healthcare institutions are the preferred sites for SCI treatment and rehabilitation, they require individuals to visit in person. Research has shown that the distance between healthcare facilities and individuals’ homes significantly affects the utilization of medical services (34). Telemedicine allows individuals to receive healthcare services at home, alleviating the inconveniences caused by transportation and time constraints, and providing real-time monitoring and rehabilitation services, which facilitate timely adjustments to treatment plans and improve rehabilitation outcomes (35). Additionally, in this systematic review, half of the studies (25, 26, 29, 32) involved individuals with SCI using web-based or app-based online learning. Telemedicine platforms typically offer health education and self-management resources, enabling individuals to learn how to better manage their condition under the guidance of their physicians, thereby enhancing their initiative and enthusiasm for rehabilitation (36). Individuals can also more easily access psychological support services (37), which is crucial for those with SCI who may face psychological stress and social barriers in addition to physical challenges. Overall, telemedicine provides more convenient, comprehensive, and personalized healthcare services for individuals with SCI, helping to improve their QoL and rehabilitation outcomes.

Subgroup analysis showed that within 3 months, telemedicine significantly improved the QoL of individuals with SCI compared to the control group. However, at 12 months, there was no significant difference in QoL between the groups. A possible reason for this discrepancy is that the novelty and convenience of telemedicine might initially motivate individuals with SCI who have not previously received such services to actively participate in the intervention, leading to better outcomes. Over time, however, psychological fatigue may set in, reducing adherence to the rehabilitation plan and diminishing the improvement in QoL. Furthermore, the included studies had a wide range of evaluation periods, lacking consistent data between 3 to 12 months, and only two studies provided data on QoL at 12 months. The small sample size and limited representativeness may affect the study’s results. Future research should include long-term follow-up studies to determine the long-term effects of telemedicine on the QoL of individuals with SCI.

#### 4.1.2 Effectiveness of Telemedicine on Depression in Individuals with SCI

According to a World Health Organization study (38), 2 to 3 out of every 10 individuals with SCI exhibit significant signs of depression. However, since depression and other psychological emotions are often considered common symptoms following SCI, they do not receive sufficient attention from medical personnel and family members (39). The results of this study showed that telemedicine has a significantly better effect on improving depression in individuals with SCI compared to standard care. However, subgroup analysis indicated that the improvement in depression scores was minimal for intervention durations of less than 6 months, with significant improvements observed only after 6 months of telemedicine intervention. People with SCI often face numerous life challenges, including physical limitations and social changes, which foster negative emotions. The development of depression can be influenced by various factors, including individual differences, social support, and the severity of the condition (40).While telemedicine provides convenience, the rehabilitation process for individuals with SCI is slow and challenging. Under these circumstances, the improvement of depressive symptoms may require long-term, continuous intervention. After receiving telemedicine services for six months, individuals with SCI showed significantly better improvement in depression compared to standard care. These findings align with a meta-analysis indicating that telemedicine interventions showed no significant improvement in depression symptoms in the early stages, but significant alleviation of symptoms was observed after 6 months of intervention (41). Additionally, A review (42) indicated that the effectiveness of telemedicine in improving depressive symptoms is nearly equivalent to that of traditional face-to-face therapy, making telemedicine a high-quality alternative for individuals with SCI. Considering these factors, the application of telemedicine in the treatment of depression among individuals with SCI holds substantial value and has significant potential for broader adoption. Over time, understanding the sustainability of treatment effects and potential intervention mechanisms will help provide a more comprehensive evaluation of telemedicine’s role in mental health.

#### 4.1.3 Effectiveness of Different Forms of Telemedicine on QoL and Depression in Individuals with SCI

Various telehealth interventions exhibit differing levels of effectiveness in improving QoL and reducing depression among individuals with SCI. Telephone counseling demonstrated limited effectiveness in enhancing QoL or alleviating depression, likely due to its lack of interactivity and real-time feedback. Mackelprang et al. (30) reported that, although telephone counseling offered psychological support and health advice, its lack of visual stimulation and personalized guidance diminished participant engagement and constrained its efficacy. In contrast, online learning and application-based personalized interventions yielded significantly greater benefits. Chemtob et al. (25) demonstrated that video-based learning on the REACTS platform significantly enhanced participants’ life satisfaction (Hedges’ g = 0.51) and engagement in health behaviors (Hedges’ g = 0.97). Similarly, Li et al. (6) developed an integrated internet platform combining video education, online consultations, and follow-ups, which significantly improved QoL and reduced depressive symptoms. The multimedia features of online platforms, including video education and interactive animations, facilitated intuitive and engaging knowledge transmission, thereby enhancing participants’ interest in learning and motivation for rehabilitation. Additionally, these platforms often incorporate real-time data tracking and feedback, enabling healthcare teams to dynamically tailor intervention strategies to participants’ rehabilitation progress. For instance, Coulter (26) and Liu (29) found that online platforms enabled participants to log exercise data, facilitating personalized guidance that significantly improved QoL and alleviated depressive symptoms. Compared to online platforms, telephone counseling exhibited limited effectiveness, although it provided some value in psychological support. Future research should investigate the integration of various telehealth modalities, including online learning, application-based support, and video monitoring, to effectively address the diverse needs of individuals with SCI and optimize rehabilitation outcomes.

### 4.2 Secondary Outcome Indicators

The results of this study indicate that telemedicine did not significantly improve anxiety and stress in individuals with SCI. Depression, stress, and anxiety have distinct psychological mechanisms and characteristics. Depression is often associated with low mood, lack of interest, and negative emotions (43), whereas stress and anxiety are more related to stress responses and hyperarousal (44). Telemedicine interventions may be more suited to addressing symptoms of depression but may have limited effectiveness for the complex responses of stress and anxiety. Many telemedicine programs primarily focus on individuals education and psychological counseling, helping them adjust their mindset and resolve practical issues. This approach may more effectively address the core issues of depression but might lack sufficient, targeted strategies to cope with the physiological responses and emotional fluctuations associated with anxiety and stress. Moreover, the anxiety and stress experienced by individuals with SCI often stem from ongoing challenges in their daily lives, including loss of physical function, social isolation, and economic difficulties. Telemedicine alone may not effectively mitigate the impact of these triggers on individuals. Additionally, the limited number of studies included in the analysis that focused on anxiety and stress may have affected the ability to detect significant effects, contributing to the lack of observed improvements. Further research is needed to develop and evaluate more comprehensive and targeted interventions for managing anxiety and stress in individuals with SCI, potentially integrating telemedicine with other support systems to address the multifaceted nature of these emotional responses.

### 4.3 Limitations and Perspectives

This meta-analysis has several limitations that should be considered. Although the 10 included studies assessed depression and QoL related outcomes, only 5 explicitly designated them as primary outcomes, with the remaining studies focusing on other indicators, such as functional improvement, physical health, or participant satisfaction. As a result, the findings may not fully reflect the potential impact of telemedicine on depression and QoL in individuals with SCI. Additionally, sample heterogeneity—including differences in participants’ demographic characteristics, injury severity, baseline health conditions, and control group care protocols—complicates the interpretation of pooled results and reduces the comparability of findings across studies. Furthermore, the geographical concentration of studies in North America, Europe, and Australia, combined with significant variations in follow-up durations, limits both the generalizability of findings and the understanding of telemedicine’s long-term effects. Some studies conducted only short-term assessments, potentially overlooking the sustained impact of interventions, especially in resource-limited regions.

To address these biases and improve the generalizability of findings, future research should focus on refining study designs. Efforts to recruit larger and more diverse samples, including individuals from a variety of demographic backgrounds, cultural contexts, and injury severities, would improve the representativeness of findings. Standardizing care protocols for control groups across studies would minimize variability and enhance the comparability of results. Additionally, extending follow-up durations, collecting interim data at regular intervals, and employing advanced analytical techniques, such as stratified analysis or meta-regression, would enable a more systematic exploration of the relationships between intervention effects and subgroup characteristics, thereby mitigating biases arising from heterogeneity. Finally, future studies should preregister protocols and ensure transparency in reporting all outcomes, including nonsignificant findings, to minimize the risk of selective reporting bias. Additionally, researchers could consider adopting the Registered Report format, which involves peer review of the study protocol before data collection begins. This extra layer of scrutiny can further reduce biases such as selective reporting and improve the overall quality of research (45). Both preregistration and Registered Reports contribute to enhancing the reliability of scientific findings, though they do so in different ways (46).

These improvements would enable future research to more comprehensively evaluate the therapeutic potential of telemedicine for individuals with SCI and provide stronger evidence of its impact on psychological health and overall QoL.

### 4.4 Strengths

This study offers several significant strengths. Firstly, this meta-analysis focuses on individuals with SCI, a group that has been underexplored in telemedicine research. By systematically evaluating the impact of telemedicine on the quality of life and depression in people with SCI, this study addresses a critical gap in the literature. Secondly, the study adheres strictly to the Cochrane Handbook guidelines for assessing the quality of the included studies. Although the overall quality of the included studies was moderate, the rigorous methodological approach ensures the reliability and transparency of the findings. Thirdly, through subgroup analyses, this study provides key insights into the differential effects of telemedicine interventions across various time points and delivery methods, offering valuable guidance for optimizing strategies in future research and clinical practice.

## 5 Conclusion

Current evidence suggests that implementing telemedicine can improve the QoL and reduce depression in individuals with SCI to some extent. Future research should focus on long-term follow-up studies to evaluate the effectiveness of telemedicine interventions for spinal cord injury management at home. Additionally, combining various telemedicine approaches and designing more rigorous, large-scale randomized controlled trials will be crucial in providing stronger evidence to guide clinical practice.

## Data Availability

The original contributions presented in the study are included in the article/supplementary material. Further inquiries can be directed to the corresponding author.
