# Supplementary material for: The efficacy of telemedicine interventions on quality of life and depression in individuals with spinal cord injury: a systematic review and meta-analysis
Source: Front Psychiatry. 2025 Jan 23;16:1434376. doi: 10.3389/fpsyt.2025.1434376 (PMC11799231; doi:10.3389/fpsyt.2025.1434376)
Supplement: Supplementary file 1 [file Table1.docx]

Supplementary Material

**Search strategy**

#1 (telerehabilitation[MeSH Terms]) OR (telemedicine[MeSH Terms])

#2 (((((((((((telerehabilitations) OR (remote Rehabilitation)) OR (virtual Rehabilitation)) OR (tele-referral)) OR (virtual medicine)) OR (telehealth)) OR (eHealth)) OR (telecare)) OR (tele intensive care)) OR (tele ICU)) OR (mobile health)) OR (mhealth)

#3 #1 OR #2

#4 spinal cord injuries[MeSH Terms]

#5 (((((spinal cord trauma) OR (traumatic myelopathy)) OR (spinal cord transection)) OR (spinal cord laceration)) OR (post-traumatic myelopathy)) OR (spinal cord contusion)

#6 #4 OR #5

#7 randomized controlled trial[Publication Type] OR randomized[Title/Abstract]OR placebo[Title/Abstract]

#8 #3 AND #6 AND #7

| **Table1.**Basic characteristics of the study | | | | | | | | | | | |
| --- | --- | --- | --- | --- | --- | --- | --- | --- | --- | --- | --- |
| Author/year | Country | Participants | | Age | Intervention group | |  | Control Group | | Outcomes assessment time | Main outcomes (assessments) |
|  |  | T | C |  | Intervention methods | Frequency & duration |  | Intervention methods | Frequency & duration |  |  |
| Dorstyn2012 (27) | Australia | 20 | 19 | I: 53.8±16.3  C: 53.1±20.0 | Telephone counseling: the introduction of interventions, education on the psychosocial impact of SCI, education, reinforcement, and monitoring of positive coping strategies, and referral to community psychological services when needed. | Once every two weeks, each time less than 20 minutes |  | Standard care: routine individual medical follow-up and physical therapies (eg, physiotherapy, occupational therapy), in addition to a face-to-face consultation with a psychologist (D.D.) at 3 months post discharge. | Not mention | Baseline, 3m, 6m | Depression, Anxiety, Stress (DASS-21) |
| Mercier2015 (31) | USA | 53 | 53 | I: 45.8±12.1  C: 45.0±14.0 | Telephone counseling: Regular automated calls provide educational content related to depression, skincare, and the perspectives of peers and clinical experts + CareCall Resource Book | Once a week for 3 months and once every two weeks after 3 months for 3 months |  | Standard care + CareCall Resource Book | Not mention | Baseline, 6m | Depression (PHQ-9) |
| Migliorini 2016 (32) | Australia | 23 | 25 | I: 47.5±12.2  C: 52.8±12.9 | Online learning: A 10-module skills and psycho-educational program based primarily on cognitive behavioral therapy principles, but with added aspects of positive psychology and mindfulness meditation | one module per week |  | Wait-list control | Not mention | Baseline, 3m, 6m | Depression, Anxiety, Stress (DASS-21) |
| Mackelprang 2016 (30) | USA | 85 | 83 | I: 40.4±15.7  C: 42.0±16.0 | Telephone counseling: (Education, problem-solving, referral resources and support) +usual care | At 1, 2, 3 and 6 weeks and 2, 3, 4, 6, 8 and 10 months after discharge, 30-45 minutes each time |  | Usual care (standard referrals and follow-up) | Not mention | Baseline, 12m | QoL (DTS)  Depression (PHQ-9) |
| Coulter 2017 (26) | Britain | 15 | 6 | I: 51.5±13.0  C: 48.1±10.6 | Online learning: The website consists of exercise, exercise diary, advice and education sections, the physical therapist can create a personalized exercise plan, remotely view the exercise diary, and change the exercise plan | 30 minutes at least twice a week for 8 weeks |  | Usual care: consisting of self-management of their condition. If participants were currently exercising (for example, home-based exercise, gym or exercise class), they were asked to continue and to keep an exercise diary noting any exercise or activities in which they participated. | Not mention | Baseline, 8w | QoL (WHO-QOL BREF)  Depression, Anxiety (HADS) |
| Arora 2017 (24) | India， Bangladesh | 57 | 58 | I: 35.0±11.0  C: 36.0±12.0 | Telephone counseling: Get a pamphlet containing information on pressure ulcer management, call a trained healthcare professional, reinforce self-help strategies important for managing pressure ulcers, minimizing psychological stress, and increasing life engagement | 1 time per week |  | Release of information kit (pressure ulcer management at the time of recruitment, and were free to seek any help or medical assistance that they deemed appropriate or could access.) | Not mention | Baseline, 12w | QoL (EQ-5D-5L)  Depression (HADS) |
| Kryger 2019 (28) | USA | 16 | 17 | I: 37.9±13.4  C: 44.1±15.3 | Online learning: The app includes medication management, urination, and defecation schedule reminders, photo-enabled skincare tracking, mood tracking, and messaging | Not mention |  | Standard care (Intermittently attended by a doctor in an outpatient setting and followed up as needed. Nurses talk to patients on the phone offer advice and relay concerns to doctors) | Not mention | Baseline, 3m, 6m, 9m | Depression (BDI-II) |
| Chemtob 2019 (25) | Canada | 10 | 12 | A: 51.6±12.1 | Online learning: An online video chat platform conducts counseling sessions designed to foster recreational physical activity (LTPA) motivation and participation through the use of behavior change techniques and self-regulatory strategies | once a week for 1 hour each time for 8 weeks |  | Regular daily routine (Continue their daily activities and are not encouraged to increase or decrease their current level of physical activity) | Not mention | Baseline, 4w, 8w | QoL (TLSQ-11)  Depression (PHQ-9) |
| Li 2021 (6) | China | 40 | 40 | I: 57.8  C: 59.1 | Online learning: 1. Establish the individuals with TSCI patient database; 2. Conduct video health education; 3. Internet platforms push knowledge | Not mention |  | Routine discharge guidance | Not mention | Baseline, 12m | QoL (SF-36) |
| Liu 2023 (29) | China | 49 | 49 | I:40.37±12.18C:43.06±12.06 | Online learning: The APP includes discharge health education (information sheets of patients and a list for discharge education） and follow-up visits (online assessment, health education, interdis-ciplinary referral, and interaction) | 5 one-on-one online sessions were scheduled at the 2nd, 4th, 6th, 8th, and 12th weeks after discharge. |  | Routine counselling (Daily care included health education and telephone follow-up by the visiting nurse at 12 weeks after discharge) +Health education CD | Not mention | Baseline, 12w,24w | Depression (BDI-II) |

I: Intervention group, C: Control group, A: All groups, DASS-21: Depression Anxiety Stress Scale-21, PHQ-9: Patient Health Questionnaire-9, EPACT: Electronic Personal Administration of Cognitive Therapy, DTS: Delighted-Terrible Scale, WHO-QOL BREF: The World Health Organisation Quality of Life Bref Scale, HADS: The Hospital Anxiety and Depression, EQ-5D-5L: Euro Quality of Life 5-dimensional 5-level, BDI-II: Beck Depression Inventory-II, TLSQ-11: The Life Satisfaction Questionnaire-11, SF-36: The MOS 36-Item Short Form Health Survey

**Table2.** Participant Characteristics and Study Focus in Telemedicine Interventions for SCI

| Author/year | | Research Focus | | | Injury Duration | Injury Level | | Injury Etiology | |
| --- | --- | --- | --- | --- | --- | --- | --- | --- | --- |
| Dorstyn2012 (27) | | Evaluates the impact of telephone counseling on mental health and life satisfaction in recently injured SCI patients | | | Recently injured, less than 1 year | Paraplegia: 62%; Tetraplegia: 38% | | Traumatic: 56%; Non-traumatic: 44% | |
| Mercier2015 (31) | | Explores the impact of the CareCall system on health management among individuals with SCI | | | Average 11.8 years | Cervical (C1-C8): 48.1%; Thoracic (T1-T12): 43.4%; Lumbar (L1-L2): 5.7% | | Not specified | |
| Migliorini 2016 (32) | | Assesses the effects of ePACT electronic cognitive behavioral therapy on depressive symptoms and life satisfaction in individuals with SCI | | | Intervention group: 11.4 years, Control group: 19.8 years | Complete tetraplegia: 3% (intervention), 8% (control); Incomplete tetraplegia: 53% (intervention), 8% (control); Complete paraplegia: 15% (intervention), 28% (control); Incomplete paraplegia: 24% (intervention), 52% (control) | | Includes both traumatic and non-traumatic injuries | |
| Mackelprang 2016 (30) | | Studies the impact of telephone consultations on health service use and mental health in individuals with SCI | | | Recently injured (within the first year post-rehabilitation) | Cervical (C1-C4): 20%; Cervical (C5-C8): 13%; Paraplegia: 28%; AIS D (incomplete): 38% | | Motor vehicle accident: 35%; Violence: 8%; Sports/recreation: 14%; Falls/struck by object: 37%; Other: 5% | |
| Coulter 2017 (26) | | Assesses online physical therapy’s impact on health and satisfaction in individuals with SCI | | | Intervention group: 13 years (SD 11.6 years), Control group: 15.7 years (SD 9.7 years) | Cervical (C1-C4): 6% (intervention), 13% (control); Cervical (C5-C8): 25% (intervention), 63% (control); Thoracic-sacral (T1-S5): 69% (intervention), 25% (control) | | Complete injury: 44% (intervention), 63% (control); Incomplete injury: 56% (intervention), 38% (control) | |
| Arora 2017 (24) | | Examines the effectiveness of telephone-based intervention for pressure injury management in individuals with SCI in low- and middle-income countries | | | Average 7 years (SD 13 years) | Upper cervical (C1-C4): 2% (intervention), 7% (control); Lower cervical (C5-C8): 19% (intervention), 22% (control); Upper thoracic (T1-T9): 28% (intervention), 23% (control); Lower thoracic (T10-L1): 35% (intervention), 38% (control); Lumbar-sacral (L2-S5): 3% (intervention), not reported in control group | | Motor vehicle accidents: 53% (intervention), 38% (control); High falls: 30% (intervention), 32% (control); Other: 17% (intervention), 25% (control) | |
| Kryger 2019 (28) | | Evaluates the iMHere mHealth system’s impact on health and psychosocial outcomes in individuals with SCI | | | Intervention group: 9.9 years (SD 8 years), Control group: 13.5 years (SD 11 years) | Paraplegia: 58% (intervention), 53% (control); Tetraplegia: 42% (intervention), 47% (control) | | Complete injury: 47% (intervention), 63% (control); Incomplete injury: 53% (intervention), 37% (control) | |
| Chemtob 2019 (25) | | Assesses the impact of self-determination theory-based telehealth intervention on leisure physical activity and quality of life in individuals with SCI | | | Minimum injury duration of 1 year | Limited to paraplegia, below cervical level | | Etiology not reported | |
| Li 2021 (6) | | Explores the effect of an online family care model on functional impairment, complications, and quality of life in traumatic individuals with SCI | | | Includes 1-year follow-up post-discharge | Cervical, thoracic, and lumbar injuries | | Traumatic, including motor vehicle accidents and falls | |
| Liu 2023 (29) | | Evaluates the effects of an APP-based self-management intervention on depression in community-dwelling individuals with SCI | | | Injury duration of 2 years or less | Cervical: 29.6%, Thoracic: 46.9%, Lumbar: 23.5%; Includes both complete and incomplete injuries | | Primarily traumatic (90.8%) | |
| **Table 3.** Summary of Included Studies: Primary and Secondary Outcome Measures | | | | | | | | | |
| First Author/ Year | | Sample Size and Population | Primary Purpose | | | Primary Outcome | | Secondary Outcome | |
| Dorstyn2012 (27) | | 48 , SCI | To evaluate the impact of remote psychological interventions on emotional regulation in individuals with SCI | | | Depression, anxiety, stress | | Emotional health and social support | |
| Mercier2015 (31) | | 106, SCI  36, MS | To assess the effectiveness of telemedicine in individuals with SCI and MS | | | Depression, skin integrity, healthcare utilization, participation, satisfaction | | Quality of life | |
| Migliorini 2016 (32) | | 48 , SCI | To evaluate the impact of remote cognitive behavioral therapy on emotional health and quality of life in individuals with SCI | | | Emotional health, life satisfaction | | Depression, anxiety, stress | |
| Mackelprang 2016 (30) | | 100 , SCI | To assess the impact of telephone counseling on healthcare utilization and mental health in individuals with SCI | | | Healthcare utilization | | Depression, quality of life, current health status, subjective health | |
| Coulter 2017 (26) | | 150 , SCI | To assess the effectiveness and participant satisfaction with remote rehabilitation training in individuals with SCI | | | Motor function, quality of life | | Depression, anxiety | |
| Arora 2017 (24) | | 120 , SCI | To evaluate the effectiveness of remote telephone intervention on pressure ulcer healing in individuals with SCI | | | Pressure ulcers | | Pain reduction, depression | |
| Kryger 2019 (28) | | 75, SCI | To assess the effectiveness of video-based cognitive behavioral therapy on mental health in individuals with SCI | | | Independence, depression, quality of life | | N/A | |
| Chemtob 2019 (25) | | 50 , SCI | To assess the satisfaction, physical activity, and quality of life improvements from telemedicine in individuals with SCI | | | Psychological need satisfaction | | Physical activity, quality of life, depression, daily activity participation | |
| Li 2021 (6) | | 60 , SCI | To explore the application of a network-based home care model in individuals with SCI | | | Complications incidence, functional disability, quality of life | | N/A | |
| Liu 2023 (29) | | 500 , SCI | To evaluate the impact of an APP-based self-management intervention on depression and anxiety in individuals with SCI | | | Depression, anxiety | | Life quality change assessment | |
